# Supplementary material for: Effectiveness of interventions aimed at reducing HIV acquisition and transmission among gay and bisexual men who have sex with men (GBMSM) in high income settings: A systematic review
Source: PLoS One. 2022 Oct 19;17(10):e0276209. doi: 10.1371/journal.pone.0276209 (PMC9581368; doi:10.1371/journal.pone.0276209)
Supplement: S1 Appendix — (DOCX) [file pone.0276209.s002.docx]

# Appendix 1

**MEDLINE (OVID)**

1 exp HIV/

2 exp HIV Infections/

3 hiv.mp. [mp=title, abstract, original title, name of substance word, subject heading word, keyword heading word, protocol supplementary concept word, rare disease supplementary concept word, unique identifier, synonyms]

4 human immunodeficiency virus.mp. [mp=title, abstract, original title, name of substance word, subject heading word, keyword heading word, protocol supplementary concept word, rare disease supplementary concept word, unique identifier, synonyms]

5 1 or 2 or 3 or 4

6 Homosexuality, Male/

7 Homosexuality/

8 Bisexuality/

9 homosexual*.mp. [mp=title, abstract, original title, name of substance word, subject heading word, keyword heading word, protocol supplementary concept word, rare disease supplementary concept word, unique identifier, synonyms]

10 bisexual*.mp. [mp=title, abstract, original title, name of substance word, subject heading word, keyword heading word, protocol supplementary concept word, rare disease supplementary concept word, unique identifier, synonyms]

11 gay.mp. [mp=title, abstract, original title, name of substance word, subject heading word, keyword heading word, protocol supplementary concept word, rare disease supplementary concept word, unique identifier, synonyms]

12 msm.mp. [mp=title, abstract, original title, name of substance word, subject heading word, keyword heading word, protocol supplementary concept word, rare disease supplementary concept word, unique identifier, synonyms]

13 men who have sex with men.mp. [mp=title, abstract, original title, name of substance word, subject heading word, keyword heading word, protocol supplementary concept word, rare disease supplementary concept word, unique identifier, synonyms]

14 6 or 7 or 8 or 9 or 10 or 11 or 12 or 13

15 5 and 14

16 randomized controlled trial.pt.

17 controlled clinical trial.pt.

18 randomized.ab.

19 placebo.ab.

20 drug therapy.fs.

21 randomly.ab.

22 trial.ab.

23 groups.ab.

24 16 or 17 or 18 or 19 or 20 or 21 or 22 or 23

25 exp animals/ not humans.sh.

26 24 not 25

27 15 and 26

28 limit 27 to yr="2012 -Current"
