# Supplementary material for: Effectiveness of interventions aimed at reducing HIV acquisition and transmission among gay and bisexual men who have sex with men (GBMSM) in high income settings: A systematic review
Source: PLoS One. 2022 Oct 19;17(10):e0276209. doi: 10.1371/journal.pone.0276209 (PMC9581368; doi:10.1371/journal.pone.0276209)
Supplement: S2 Appendix — (DOCX) [file pone.0276209.s003.docx]

# Appendix 2

Andorra

Antigua and Barbuda

Aruba

Australia

Austria

Bahamas, The

Bahrain

Barbados

Belgium

Bermuda

British Virgin Islands

Brunei Darussalam

Canada

Cayman Islands

Channel Islands

Chile

Curaçao

Cyprus

Czech Republic

Denmark

Estonia

Faroe Islands

Finland

France

French Polynesia

Germany

Gibraltar

Greece

Greenland

Guam

Hong Kong SAR, China

Hungary

Iceland

Ireland

Isle of Man

Israel

Italy

Japan

Korea, Rep.

Kuwait

Latvia

Liechtenstein

Lithuania

Luxembourg

Macao SAR, China

Malta

Monaco

Netherlands

New Caledonia

New Zealand

Northern Mariana Islands

Norway

Oman

Palau

Poland

Portugal

Puerto Rico

Qatar

San Marino

Saudi Arabia

Seychelles

Singapore

Sint Maarten (Dutch part)

Slovak Republic

Slovenia

Spain

St. Kitts and Nevis

St. Martin (French part)

Sweden

Switzerland

Taiwan, China

Trinidad and Tobago

Turks and Caicos Islands

United Arab Emirates

United Kingdom

United States

Uruguay

Virgin Islands (U.S.)
